# Effects of transcutaneous vagus nerve stimulation on chronic low back pain: a systematic review

**DOI:** 10.1186/s12891-024-07569-w

**Published:** 2024-06-26

**Authors:** Lama Eid, Mina George, Doaa A. Abdel Hady

**Affiliations:** 1 Faculty of Physical Therapy, Deraya University, Minia, Egypt; 2 Department of Physical Therapy for Women’s Health, Faculty of Physical Therapy, Deraya University, Minia, Egypt

**Keywords:** Physiotherapy, Rehabilitation, Transcutaneous vagus nerve stimulation, Chronic low back pain

## Abstract

**Background:**

Chronic low back pain (CLBP) is a frequent disease. It is a critical health concern that can influence functional capacity by restricting living activities.

**Objectives:**

The current study is to investigate the effects of transcutaneous vagus nerve stimulation (TVNs) in the management of CLBP.

**Methods:**

We searched the databases on Google Scholar, PubMed, Web of Science, Cochrane, and Pedro for randomized clinical trial (RCT) studies published in any language that looked at the effectiveness of TVNs in people with chronic LBP. The inclusion criteria were PICO. Participants in the research were people (≥ 18 years) diagnosed with persistent low back pain for more than 3 months. Study quality was assessed using Cochrane ROB 2.

**Results:**

Our database search found 1084 RCT. A number of studies that were not necessary for the issue were removed, and the overall outcome was six trials. Risk of bias (ROB) evaluations at the study level (derived from outcomes) are reported. In the six studies, two (33.3%) had an overall uncertain ROB (i.e., some concerns), whereas one (16.7%) had a high overall ROB. Three trials (50%) had a low overall RoB.

**Conclusion:**

There is still no evidence to support the use of transcutaneous vagus nerve stimulation as a viable therapeutic rehabilitation strategy. Therefore, we recommend high-quality trials and long-term follow-up to evaluate disability, quality of life, and pain outcomes in these patients.

**Supplementary Information:**

The online version contains supplementary material available at 10.1186/s12891-024-07569-w.

## Introduction

Low back pain (LBP) is described as discomfort present between the costal line and the gluteal [1]. Chronic low back pain (CLBP) is a widespread and frequently debilitating musculoskeletal disorder [2]. CLBP, which normally lasts at least 12 weeks, is estimated to be the major cause of disability globally and appears as the primary issue for well-being [1]. In addition to increasing disability, low back discomfort reduces people's productivity and overall quality of life. According to the 2017 Global Burden of Disease Study, low back pain is one of the top 10 most common causes of disability [3]. In developed nations, the prevalence of LBP varies from 60 to 70%. Only 39–76% of patients fully recover from an acute bout of pain, implying that a significant proportion of them develop a chronic illness [4–6]. The prevalence grows and peaks between the ages of 35 and 55. It has a significant influence on both people and society [7]. Analgesics, nonsteroidal anti-inflammatory drugs, steroids, relaxing medications, and antidepressants are all options for treatment. Non-medical alternative therapies include education, therapeutic exercise, manual manipulation therapy, traction, orthotics, transcutaneous electrical nerve stimulation (TENS), therapeutic massage, and meditation [3]. Transcutaneous vagus nerve stimulation (tVNS) has been researched for its advantages in patients with fibromyalgia, migraine, and cluster headache. Several investigations of individuals with epilepsy and depression indicated that LBP patients reported less pain and had a higher quality of life [7]. The hypothalamus-pituitary-adrenal (HPA) axis is responsible for reducing pain at the peripheral level, affecting central and peripheral sensitization through TNF-α, and playing a role in the limbic area that impacts psychological factors [8].

TVNS is one of the techniques being explored and used to treat chronic pain. The efficacy of this medication has been demonstrated in fibromyalgia and migraine [9]. Several trials on epileptic and depressed individuals found that tVNS alleviated their discomfort [10]. The FDA recommends stimulating the auricular branch vagus nerve (ABVN) in the conchae, cymba conchae, and tragus at a frequency of 20–30 Hz. Several studies have demonstrated safety and acceptability over the past decade [11].

TVNS reduces chronic pain through a pain-modulating action on serotonergic and noradrenergic pathways, as evidenced by activity in the locus coeruleus and nucleus raphe in functional magnetic resonance imaging (fMRI). TVNS’s anti-inflammatory effect was discovered via the hypothalamus-pituitary-adrenal (HPA) axis, an anti-inflammatory cholinergic mechanism responsible for reducing pain at the peripheral level, affecting central and peripheral sensitization via the TNF-a mechanism, and playing a role in the limbic area, which influences psychological factors [12]. Several systematic studies have been conducted on manual treatments such as spinal manipulation, the muscular energy method, mobilization [13–17], and acupuncture as methods for treating backaches [17].

Regarding the absence of understanding about the effectiveness of VNS, the goal of this systematic review of RCTs was to evaluate the effects of TVNs for chronic nonspecific LBP patients in terms of pain intensity, functional ability, and overall quality of life.

## Materials and methods

### Design of study

The present research followed the PRISMA guidelines regarding systematic reviews [18]. Using the methodology suggested by the Cochrane Collaboration's recommendations for performing an overview of systematic reviews [19].

### Eligibility criteria

For this systematic review, we selected only randomized controlled trials (RCTs) published in any language that investigated the efficacy of TVNs in people with chronic LBP. The inclusion criteria were PICO (patients, intervention, comparator, and outcome) RCTs evaluating the efficacy of TVNS for chronic non-specific LBP. No language restrictions apply.

Articles were created between 2000 and 2023. Provide detailed, unique articles that extract crucial information from research findings.

We omitted that criteria. Studies on individuals under the age of 18 with CLBP lasting less than three months. Studies that did not look at the severity of low back pain.Trials that fail to provide results or offer insufficient data. It includes methods, suggestions, editorials, book chapters, letters to editors, reviews, and meta-analyses. Animal research. Alternative approaches to conducting randomized controlled trials. Patients with prior back surgery, lumbar disc herniation, spinal abnormalities, neuromusculoskeletal issues, rheumatoid arthritis, osteoporosis, or poster presentations for studies were discontinued.

#### Population

Participants in the research were people (≥18 years) diagnosed with persistent low back pain for more than 3 months by a doctor.

#### Intervention

The intervention was tVNS, which was compared to exercise therapy or a control group.

### Comparator

No limitations were set for comparator interventions.

**Outcomes**:

**Primary outcomes**

-pain

-functional capacity

**Secondary outcomes**

**-**endurance

-quality of life

-disability

- C-reactive protein

### Search criteria and strategy

This systematic review was done according to the preferred reporting items for systematic reviews [20]. We searched PubMed, Cochrane Library, Scopus, Pedro, Web of Science, and Google Scholar. We utilized the search strategies of ("vagus nerve stimulation" OR VNS) AND ("chronic low back pain" OR "nonspecific low back pain" OR "mechanical low back pain") on the data bases previously mentioned as the main search strategies, as well as (auricular nerve stimulation on chronic low back pain) and (TVNs on nonspecific low back pain). Only studies were obtained and examined by two separate reviewers, who then compared and supplemented the findings to remove duplicate material using Endnote's checking feature. The author (L.E.) examined the records, and the author (M.G.) checked the same data for precision, all under the supervision of the author (D.A.). After duplication, prospective articles were selected based on their abstracts. Relevant information was retrieved from the full text of the chosen publications. Additional papers were discovered by manual searches of referenced references (snowball referencing). Disagreements in the assessments were handled in a consensus dialogue after comparing discrepancies between assessors and were discussed among the whole research group guided by DA, which was carried out from September 2023 to February 2024.

### Data collection

**Fig. 1:**
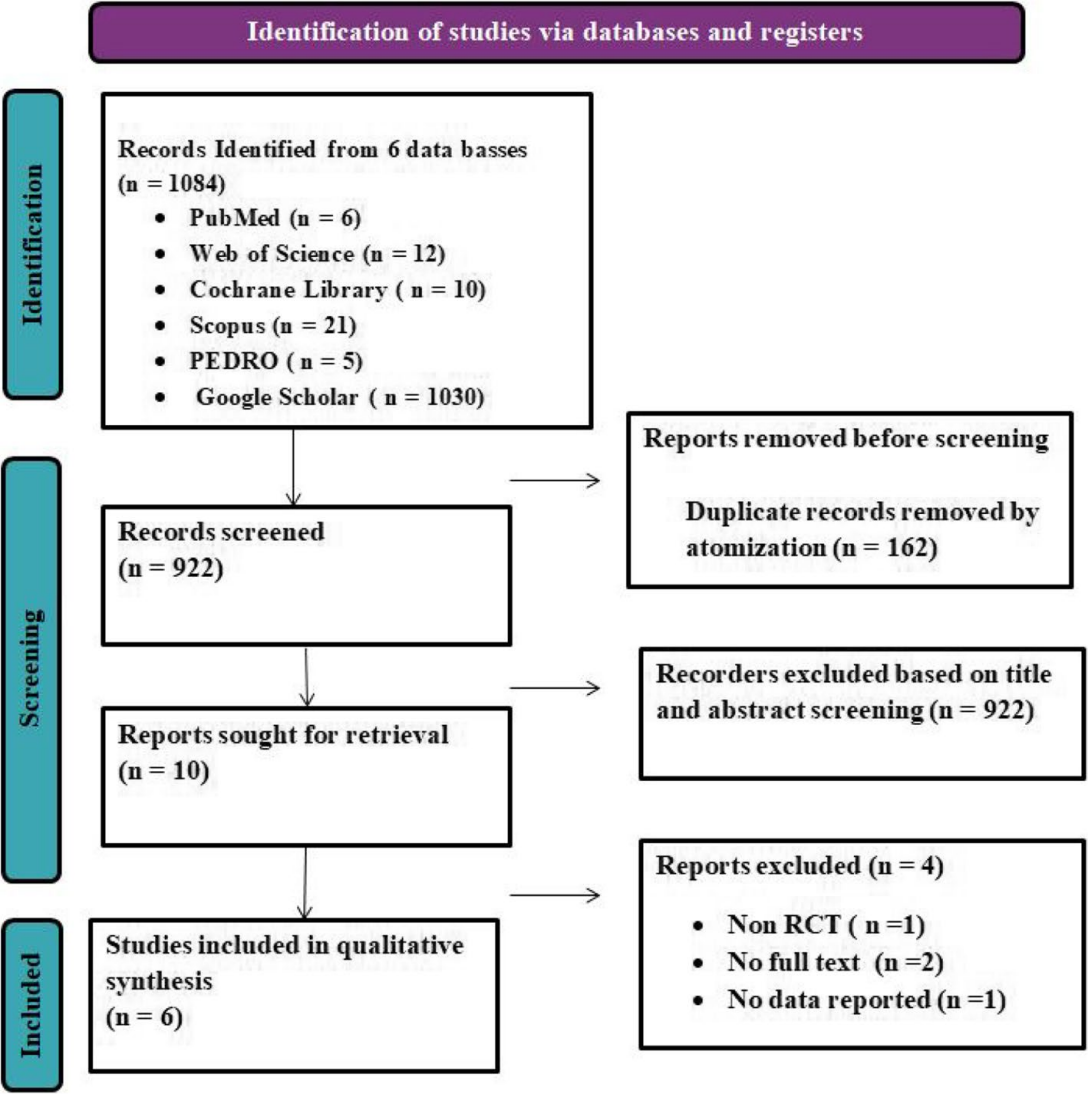
PRISMA for chart illustrating the process of inclusion of articles in the study

All data relevant to the evidence synthesis were extracted by authors (M.G) and author (L.E) Fig. 1.

### Data extraction and synthesis

Two judges prepared a uniform data extraction sheet. The data was extracted using a standard Excel spreadsheet. outlines the essential characteristics of the included studies. Authors, publication year, sample size, age, gender, participants, BMI, number of treatments, therapy duration, pain assessment approach, and significant findings. The authors were contacted when data collection was required (Tables 1, 2, 3, 4, 5 and 6).

**Table 1:** Characteristics of included studies

Study																
	Secondary outcomes															
	AVERAGE (SF36)								Emotional role difficulty (SF -36)							
	Intervention group				Control group				Intervention group				Control group			
	pre treatment		Post treatment		pre treatment		Post treatment		pre treatment		Post treatment		pre treatment		Post treatment	
	Mean	SD	Mean	SD	Mean	SD	Mean	SD	Mean	SD	Mean	SD	Mean	SD	Mean	SD
(Halim et al., 2023) [32]	69.41	11.02	85.28	9.12	69.94	11.33	82.01	11.18	78.79	34.23	87.88	22,47	69.71	27.7	81.82	31.13

Study	Outcomes															
	Secondary outcomes															
	Vitality(SF36)								Mental Health (SF -36)							
	Intervention group				Control group				Intervention group				Control group			
	pre treatment		Post treatment		pre treatment		Post treatment		pre treatment		Post treatment		pre treatment		Post treatment	
	Mean	SD	Mean	SD	Mean	SD	Mean	SD	Mean	SD	Mean	SD	Mean	SD	Mean	SD
(Halim et al., 2023) [32]	75	11.4	86.36	13.8	72.72	12.32	82.27	16.02	89.45	8.99	94.18	6.28	80.72	11.97	89.09	13.27

**Table 2:** Characteristics of included studies

Study	outcomes															
	Primary out comes															
	Pain (NPRS)								pain (SF-36)							
	intervention group				controle grop				Intervention group				control group			
	pre treatment		Post tretment		pre treatment		Post treatmen		Pre treatment		Post treatment		Pre treatment		Post treatment	
	Mean	SD	Mean	SD	Mean	SD	Mean	SD	Mean	SD	Mean	SD	Mean	SD	Mean	SD
(Halim et al., 2023) [30]	5.45	1.12	1.73	1.27	5.82	1.07	3.27	1.61`								
(Halim et al., 2023) [32]									50.45	17.6	86,59	9.7	69.31	13.92	83.4	12.26

**Table 3:** Characteristics of included studies

Study	Outcomes																							
	primary outcomes																							
	Function capicity (TUG)								Function capicity (FTSST)								Functional Capacity/ Physical Function (Sf36)							
	intervention group				controle grop				intervention group				controle grop				Intervintion				Control group			
	pre treatment		Post treatment		pre treatment		Post treatment		pre treatment		Post treatment		pre treatment		Post treatment		pre treatment		Post treatment		pre treatment		Post treatment	
	Mean	SD	Mean	SD	Mean	SD	Mean	SD	Mean	SD	Mean	SD	Mean	SD	Mean	SD	Mean	SD	Mean	SD	Mean	SD	Mean	SD
(Halim et al., 2023)																	65	20.24	79.54	19.67	76.36	12.86	88.18	9.81
(Uzlifatin et al ., 2023)	10.13	2.68	7.76	1.46	9.23	2	8.71	3.13	18.06	9.2	12.33	2.42	12.12	4.05	12.17	3.01

**Table 4:** Characteristics of included studies

Study	Outcomes															
	Secondary outcomes															
	AVERAGE (SF36)								Emotional role difficulty (SF -36)							
	Intervention group				Control group				Intervention group				Control group			
	pre treatment		Post treatment		pre treatment		Post treatment		pre treatment		Post treatment		pre treatment		Post treatment	
	Mean	SD	Mean	SD	Mean	SD	Mean	SD	Mean	SD	Mean	SD	Mean	SD	Mean	SD
(Halim et al., 2023) [32]	69.41	11.02	85.28	9.12	69.94	11.33	82.01	11.18	78.79	34.23	87.88	22,47	69.71	27.7	81.82	31.13

Study	Outcomes															
	Secondary outcomes															
	Vitality(SF36)								Mental Health (SF -36)							
	Intervention group				Control group				Intervention group				Control group			
	pre treatment		Post treatment		pre treatment		Post treatment		pre treatment		Post treatment		pre treatment		Post treatment	
	Mean	SD	Mean	SD	Mean	SD	Mean	SD	Mean	SD	Mean	SD	Mean	SD	Mean	SD
(Halim et al., 2023) [32]	75	11.4	86.36	13.8	72.72	12.32	82.27	16.02	89.45	8.99	94.18	6.28	80.72	11.97	89.09	13.27

**Table 5:** Characteristics of included studies

Study	Outcomes															
	secondary outcomes															
	Social Functionality (SF36)								Physical Role Difficulty (SF36)							
	Intervention group				Control group				Intervention group				Control group			
	pre treatment		Post treatment		pre treatment		Post treatment		pre treatment		Post treatment		pre treatment		Post treatment	
	Mean	SD	Mean	SD	Mean	SD	Mean	SD	Mean	SD	Mean	SD	Mean	SD	Mean	SD
[32](Halim et al., 2023)	78.4	17.75	93.18	10.25	71.59	14.88	79.54	15.07	61.36	34.21	77.27	28.4	56.81	37.23	77.27	30.52

**Table 6:** Characteristics of included studies

Study	Outcomes																							
	secondary outcomes																							
	Endurance (BST)								crp								Disability (RMDQ)							
	Intervention group				Control groupsssss				Intervention group				Control group				Intervention group				Control group			
	pre treatment		Post treatment		pre treatment		Post treatment		pre treatment		Post treatment		pre treatment				pre treatment				pre treatment		Post treatment	
	Mean	SD	Mean	SD	Mean	SD	Mean	SD	Mean	SD	Mean	SD	Mean	SD	Mean	SD	Mean	SD	Mean	SD	Mean	SD	Mean	SD
(Uzlifatin et al ., 2023) [31]																	9.45	4.44	2.18	2.71	10.55	5.05	2.36	2.06
(kusumastuti et al., 2023) [34]	40.3	31.2	66.49	45.87	58.18	50.75	66.82	38.56																
(Uzlifatin et al ., 2023) [35]									0.22	0.13	0.39	0.3	0.21	0.18	0.22	0.18								

#### Evidence synthesis

The primary outcome for persistent low back pain was pain evaluated by NPRS [21]. Secondary outcomes included disability evaluated by RMDQ [22], back muscular endurance assessed using BST [23, 24], quality of life measured by the SF36 scale [25], and inflammatory state measured by the amount of C reactive protein functional capacity determined by both FTSST [26, 27] and TUG tests [28, 29]. All secondary outcomes were recorded in the included records. Due to the limited number of RCTs for each outcome or symptom, evidence synthesis was carried out qualitatively.

#### Characteristics of the included studies

The studies had trials with sample sizes ranging from 20 to 22. The individuals in the six research studies varied in age from 18 to 55 years, and treatments lasted around 20 minutes every day for two weeks. Highlights each study's important demographic and clinical features. Of the six studies published by the Faculty of Medicine at the University of Airlangga, Dr. Soetomo General Academic Hospital.

## Results

### Search result

There were a total of 1084 references, including 6 from PubMed, 12 from Web of Science, 10 from the Cochrane Library, 21 from Scopus, 5 from PEDRO, and 1030 from Google Scholar. Of these, 162 duplicate instances were eliminated. After evaluating the titles and abstracts of 922 studies, ten records were removed, four were deleted after following the exclusion criteria, and ten publications were included in the final qualitative analysis. A flow diagram depicts the search approach. Six RCTs were found and published in the year 2023 (Fig. 1).

### Study quality assessment

**Fig. 2:**
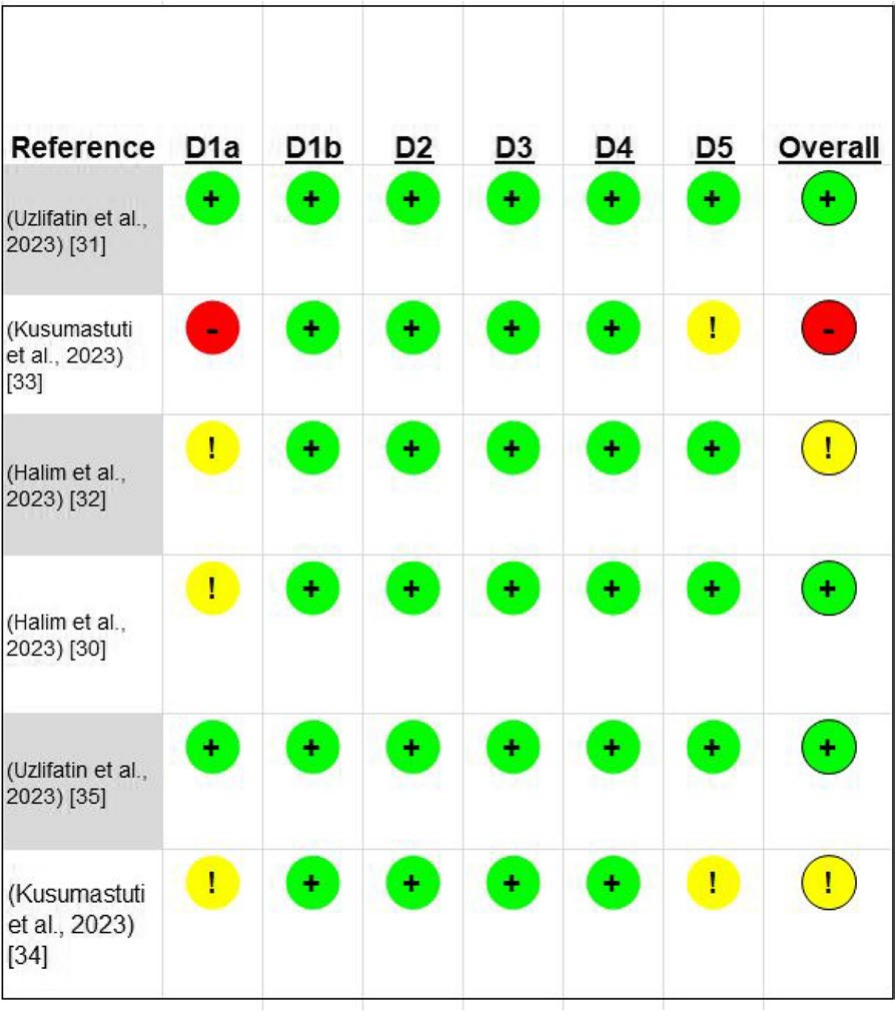
RoB assessments on study level (as derived from the outcomes) are summarized in Fig. 2 and Supplementary data appendix E. Of the 6 studies, 2 (33.3%) had an overall unclear RoB (ie, some concerns), and 1 (16.7%) a high overall RoB. 3(50%) had an overall unclear RoB (ie, some concerns), Figure 2 Risk of bias rating on an individual study level, displayed as traffic light plot for each study and/or outcome with green lights = low, yellow = unclear and/or moderate, and red = high risk of bias. The aggregate Cochrane Risk-of-bias appraisal results summary plot is displayed in the Supplementary data appendix E. Rob-2 Tool Cochrane. D1. Randomization process; D2. deviations from the intended interventions; D3. missing outcome data; D4. measurement of the outcome; D5. selection of the reported results

The Cochran RoB2 assessment form was used to evaluate the quality of the chosen research, and each study's quality of methodology was independently appraised by two researchers. Controversies between the two reviewers were addressed through discussion and consensus. The RoB2 tool offers a framework for assessing the risk of bias in the results of any randomized controlled experiment. Bias is examined across five key domains. Within each domain, RoB 2 users respond to one or more signaling questions. These responses provide evaluations of “low risk of bias”, “some concerns”, or “high risk of bias” (Fig. 2) (Table 7).

**Table 7:** Summary of methodological quality assessments based on cochrane ROB2 classification tool

Reference	Experimental	Comparator	Outcome	Result measurements	Randomization process	Timing of identification or recruitment of participants	Deviations from intended intersssventions	Mising outcome data	Measurement of the outcome	Selection of the reported result	Overall Bias
(Uzlifatin et al., 2023) [31]	TVNs	exersice therapy	dasaibility	RMDQ score	Low	Low	Low	Low	Low	Low	Low
(Kusumastuti et al., 2023) [33]	TVNs	Exersise	muscle endurance	(BST) test score	High	Low	Low	Low	Low	Some concerns	High
(Halim et al., 2023) [32]	TVNs	exersice therapy	participants’ quality of life	SF-36 score.	Some concerns	Low	Low	Low	Low	Low	Some concerns
(Halim et al., 2023) [30]	TVNs	exersice therapy	Numerical Pain Rating Scale (NPRS)	The mean NPRS	Some concerns	Low	Low	Low	Low	Low	Low
(Uzlifatin et al., 2023) [35]	TVNs	EXersise	CRP Level	CRP blood sampling level	Low	Low	Low	Low	Low	Low	Low
(Kusumastuti et al., 2023) [34]	TVNs	Exersise therapy	Functional capacity	FTSST and TUG tests	Some concerns	Low	Low	Low	Low	Some concerns	Some concerns

The majority of the included studies employed TVNS as part of a combined therapeutic approach, rendering it challenging to definitively ascertain the true efficacy of VNS in the treatment of chronic low back pain.

### Effectiveness of TVNs on pain intensity, functional capacity, quality of life, back muscle endurance, and disability for CLBP

All six trials found that TVNs were beneficial in lowering pain intensity, boosting quality of life, back muscular endurance, and functional capacity, but had no notable reduction in disability or decrease in C-reactive protein. These studies assessed the efficacy of TVNs in contrast to exercise treatment alone. The tools used for measurement were the NPRS for evaluating pain, the Roland Morris Disability Questionnaire (RMDQ) for assessing disability, the quality of life short form questionnaire (SF-36) for quality of life, the c-reactive protein level for inflammatory state, the timed up and go (TUG), and the five-time set to stand (FTSST) tests for functional capacity evaluation.

## Discussion

The intention of the research was to conduct a comprehensive evaluation of TVNs' efficacy in individuals with persistent low back pain. Several systematic studies have been conducted on manual treatments such as various exercise types, spinal manipulation, the muscular energy method, mobilization, and acupuncture as methods for treating backaches. According to the systematic reviews on the use of TVNs in LBP published so far [30–35], numerous studies employed TVNs as an element of combination therapy, complementing other physiotherapeutic effects such as kinesiotherapy, physical therapy, and other manual treatment approaches. During such protocols, in this review, patients with CLBP reported decreased pain after using the TVNs. This conclusion confirms past studies [30–35]. They also discovered that employing TVNs helped individuals with LBP experience less pain throughout their own studies. Television networks and exercise initiatives. Furthermore, the same benefits have been shown in trials utilizing brief exercise regimens combined with TVNs. Exercises were previously connected with alleviating pain and improving quality of life in both the short and long term [31]. Only one of the included studies includes at least one physical component (exercise, physical modalities) as well as one other aspect (psychological, social, or occupational) in the indicated impacts on quality of life. According to [36], there is strong evidence that TVNs have a favorable effect on pain.

A further study demonstrated that the regulation of nociception and pain perception by pVNS is highly dependent on the precise electrical stimulation program and treatment location [37, 38]. In this investigation, the stimulation amplitude was fixed, resulting in a tingling (but not painful) feeling at the stimulation site. pVNS focuses on Aβ-fibers that regulate cutaneous mechanoreception and touch sensation, avoiding activation of Aδ-fibers implicated in affective-emotional pain activities [37].

This study is based on the Cochrane method, which involves analyzing clinical RCT evidence, searching and screening the main electronic publication database for evidence-based medical research, and providing clinicians with stronger proof when making decisions to better guide clinical treatment. Future research on TVNs should use a more rigorous technique. To avoid bias, subsequent RCTs should closely comply with the CONSORT principles [39], particularly in terms of publication of research procedures and blinding. The majority of the included trials used VNS as part of a multimodal therapy strategy, making it difficult to determine the real efficacy of VNS in the treatment of persistent low back pain. We believe that conducting further high-quality RCTs will help corroborate the current findings.

### Limitations

There are limited studies available, with significant study constraints, difficulties with directness and inaccuracy, and treatment protocols (the length of tVNS sessions is only two weeks), and more research is needed to strengthen the confidence of findings. The minimal number of studies available for systematic evaluation precluded us from conducting a meta-analysis. The clinical trials included in the systematic review were all RCTs, although there were still issues with blinding and allocation concealment during implementation. Blinding and allocation concealment are critical during the implementation of RCTs since they may enhance patient score bias or the effect of participants' subjective aspects in the research. Also, 'the minimal number of studies available for systematic evaluation precluded us from conducting a meta-analysis' because meta-analyses typically involve a modest amount of research (≤ 5). Estimating between-study heterogeneity is problematic in this study. Acceptance of pre-registered protocols is a limitation of systematic review.

## Conclusion

There is still no evidence to support the use of transcutaneous vagus nerve stimulation as a viable therapeutic rehabilitation strategy. Therefore, we recommend high-quality trials and long-term follow-up to evaluate disability, quality of life, and pain outcomes in these patients.

### Supplementary Information

Supplementary Material 1.

## Data Availability

The datasets generated during and/or analyzed during the current study are available from the corresponding author on reasonable request.
